# Dynamics of pulmonary mucosal cytotoxic CD8 T-cells in people living with HIV under suppressive antiretroviral therapy

**DOI:** 10.1186/s12931-024-02859-2

**Published:** 2024-06-12

**Authors:** Yulia Alexandrova, Alexis Yero, Ronald Olivenstein, Marianna Orlova, Erwin Schurr, Jerome Estaquier, Cecilia T. Costiniuk, Mohammad-Ali Jenabian

**Affiliations:** 1 Department of Biological Sciences, Université du Québec à Montréal (UQAM), 141, Avenue President Kennedy, Montreal, QC H2X 1Y4 Canada; 2 Infectious Diseases and Immunity in Global Health Program, Research Institute of McGill University Health Centre, Montreal, QC Canada; 3 Division of Respirology, Department of Medicine, McGill University, Montreal, QC Canada; 4 Departments of Human Genetics and Medicine, McGill University, Montreal, QC Canada; 5 Centre de recherche de CHU de Québec - Université Laval Research Center, Québec City, Québec Canada; 6 Division of Infectious Diseases and Chronic Viral Illness Service, McGill University Health Centre, Montreal, QC Canada

**Keywords:** HIV, Smoking, People living with HIV (PLWH), Pulmonary immunity, CD8 T-cells, Lung, Trm, Tissue resident

## Abstract

**Background:**

Despite the success of antiretroviral therapy (ART), people living with HIV (PLWH) suffer from a high burden of pulmonary diseases, even after accounting for their smoking status. Cytotoxic CD8 T-cells are likely implicated in this phenomenon and may act as a double-edged sword. While being essential in viral infection control, their hyperactivation can also contribute to lung mucosal tissue damage. The effects of HIV and smoking on pulmonary mucosal CD8 T-cell dynamics has been a neglected area of research, which we address herein.

**Methods:**

Bronchoalveolar lavage (BAL) fluid were obtained from ART-treated PLWH (median duration of supressed viral load: 9 years; smokers: *n* = 14; non-smokers: *n* = 21) and HIV-uninfected controls (smokers: *n* = 11; non-smokers: *n* = 20) without any respiratory symptoms or active infection. Lymphocytes were isolated and CD8 T-cell subsets and homing markers were characterized by multiparametric flow cytometry.

**Results:**

Both smoking and HIV infection were independently associated with a significant increase in frequencies of total pulmonary mucosal CD8 T-cell. BAL CD8 T-cells were primarily CD69 + expressing CD103 and/or CD49a, at least one of the two granzymes (GzmA/GzmB), and little Perforin. Higher expression levels of CD103, CD69, and GzmB were observed in smokers *versus* non-smokers. The ex vivo phenotype of GzmA + and GzmB + cells revealed increased expression of CD103 and CXCR6 in smokers, while PLWH displayed elevated levels of CX3CR1 compared to controls.

**Conclusion:**

Smoking and HIV could promote cytotoxic CD8 T-cell retention in small airways through different mechanisms. Smoking likely increases recruitment and retention of GzmB + CD8 Trm via CXCR6 and CD103. Heightened CX3CR1 expression could be associated with CD8 non-Trm recruitment from the periphery in PLWH.

**Supplementary Information:**

The online version contains supplementary material available at 10.1186/s12931-024-02859-2.

## Introduction

Despite the success of antiretroviral therapy (ART), people living with HIV (PLWH) suffer from a disproportionate burden of infectious and non-infectious pulmonary diseases, suggesting that their lung immunity is not fully restored [1–3]. Moreover, smoking prevalence in PLWH is almost twice as high compared to the general population, contributing to low grade chronic systemic inflammation and decreased ART efficacy in these individuals [1, 2, 4–6]. Helleberg and others report that both all-cause and non-AIDS-related mortalities are higher among PLWH who smoke than among those who do not and that this population loses more life-years to smoking than to HIV infection itself (12.3 years *versus* 5.1 years respectively) [7]. Importantly, both smoking and HIV are independent risk factors for pulmonary complications such as lung cancer, emphysema, and Chronic Obstructive Pulmonary Disease (COPD) [1, 2, 8–10].

Within this context, lung mucosal CD8 T-cells play a critical role in controlling chronic viral infection, while potentially harming surrounding tissues and contributing to pulmonary pathologies frequently seen in PLWH [1, 2]. HIV is rapidly seeded within the lung during primary infection, where proximity of millions of alveoli provide a large surface area for cell-to-cell viral spread [1, 11, 12]. Our team have also previously shown that HIV-DNA persists within pulmonary CD4 and double negative T-cells despite a decade of ART [13, 14]. Importantly, CD8 T-cells are critical for HIV control. Animal studies have shown that CD8 T-cell depletion in SIV-infected Rhesus Macaques treated with short-term ART, leads to increased plasma viremia, which is reversible with CD8 T-cell repopulation [15]. CD8 T-cells may accumulate in the lung upon HIV seeding within that tissue. In fact, asymptomatic PLWH frequently develop “CD8 T-cell alveolitis” characterized by accumulation of HIV-specific pulmonary T-cells, which is associated with respiratory symptoms and worse clinical outcomes [1, 16, 17]. While CD8 T-cells are crucial for the clearance of HIV-infected cells and opportunistic infections, chronic inflammation and antigen stimulation result in impairment of their antiviral functions in PLWH [1, 2, 17]. Although some of these functions do recover in the peripheral blood after ART initiation, this is not the case for pulmonary CD8 T-cells [18, 19]. In the context of other chronic pulmonary viral infections and lung cancers, CD8 T-cells also lose their cytotoxic activity during late-stage differentiation due to immune exhaustion [20–23], increasing the risk of opportunistic infections and malignancies [24, 25]. Moreover, these cells might induce excessive expansion of other CD8 T-cells in the vicinity via T-cell receptor independent mechanisms, known as “bystander activation” [26, 27]. Collectively, chronic immune activation during HIV infection is one of the likely mechanisms driving accumulation of functionally impaired CD8 T-cells displaying reduced proliferation, poor effector functions, and high expression of inhibitory receptors in the lung as reported by our team and others [19, 26, 28–30].

Cigarette smoke can have both pro- and anti-inflammatory effects by inducing the production of TNF-α, IL-1β, IL-6, IL-8, and monocyte chemoattractant protein-1 [31, 32]. However, other studies indicate that nicotine, the most addictive component of tobacco, does the opposite, where it can decrease levels of IL-6, IL-8, and IL-10 through the engagement of α7 nicotinic acetylcholine receptor [33–35]. Increased production of pro-inflammatory cytokines in smokers has a significant effect on their BAL fluid immune cell concentration and composition: BAL fluid from smokers has increased number of neutrophils, macrophages and CD8 T-cells compared to non-smokers [36, 37]. Subsequently, BAL CD4/CD8 T-cell ratio in smokers is reduced. Studies also show that these CD8 and CD4 T-cells are skewed towards a type 1 phenotype (Tc1 and Th1 cells respectively), which are IFN-γ producing cells that help fight intracellular infections but are also capable of causing tissue destruction in COPD patients [38–40]. We recently demonstrated that pulmonary CD8 T-cells show lower ex vivo perforin expression, lower ability to upregulate degranulation upon in vitro stimulation, and limited HIV-specific killing capacity compared with blood CD8 T-cells regardless of HIV or smoking status [30]. On the one hand, this could potentially contribute to a suboptimal anti-HIV immune response and the establishment of viral reservoirs within the lungs. On the other hand, this low cytotoxic profile might be part of a normal physiological cell state that acts as a safeguard against undue tissue damage to a vital organ. Compared to the blood, pulmonary mucosa is exposed to high levels of oxygen, airborne particles, and microbes. Furthermore, nutrients are scarce in that tissue, which in turn, can affect CD8 T-cell metabolism and function [41, 42]. Transcriptomic analysis studies in murine models also report that, in contrast to cells from the pulmonary interstitium and the circulation, CD8 T-cells from the airways display lower expression of genes involved in cytotoxic function [41]. This property of tissue-resident memory CD8 T-cells (CD8 Trm) during homeostatic conditions might be reversed in context of chronic inflammation, resulting in tissue destruction. In chronic inflammatory lung conditions, like smoking and COPD, increased CD8 T-cell cytotoxicity and reduced apoptosis in the small airways can compromise the integrity of the mucosal barrier by killing alveolar epithelial cells via perforin, granzyme-B, and TNF-α [43–50]. Notably, we have previously reported significant reductions in proportions of senescent pulmonary CD28-CD57 + CD8 T-cells in smoking PLWH, which was not observed in seronegative controls, suggesting differential dynamics of CD8 T-cells in these study participants [30].

Overall, CD8 T-cells’ cytotoxic functions are needed for pathogen clearance and infection control but can wreak havoc if left unchecked. In the context of pulmonary mucosa, we do not yet know at what differentiation or tissue migration stage CD8 T-cells begin to lose their cytotoxic functions. In this study, we aimed to investigate pulmonary CD8 T-cell dynamics in PLWH and uninfected individuals, while accounting for the impact of tobacco smoking. Across four different study groups, we performed a comprehensive flow-cytometric characterization of CD8 T-cell phenotypes based on their expression of effector proteins, tissue-residency markers, and chemokine receptors involved in T-cell migration.

## Methods

### Study population

ART treated (suppressed VL ≥ 1 year and CD4 count ≥ 350 cells/mm3) smokers and non-smokers in addition to HIV-uninfected smokers and non-smokers were recruited at the McGill University Health Centre (MUHC). All participants underwent bronchoscopies for research purposes only. Participants underwent spirometric testing several weeks prior to bronchoscopy to ensure the absence of any undiagnosed obstructive airflow disease. The participants were also screened for the absence of active pulmonary infection and any other acute pulmonic process. Participants were labelled as smokers if they smoked at least 1 tobacco cigarette daily. 50–100 ml of BAL fluid were obtained via bronchoscopies performed by a respirologist and matched 40 ml blood samples were collected by venipuncture the same day.

### Lymphocyte isolation

As per a recent protocol optimized and published by our team, BAL fluid was centrifuged within the first hour following the bronchoscopy to isolate the cells and remove any BAL liquid. Lymphocytes were isolated from both blood and BAL as previously described [13, 30, 51]. All samples underwent cryopreservation prior to subsequent flow cytometric analysis in batch.

### Flow cytometry

Live pulmonary CD8 T-cells were characterized ex vivo using CD49a/CD69/CD103 (Trm markers), CD45RA/CCR7 (T-cell memory subsets), CXCR3/CXCR6 (CD8 T-cell migration from the interstitium), CX3CR1/KLRG1 (CD8 T-cell migration from peripheral circulation) [52–57], and granzyme-A/granzyme-B/perforin (cytotoxicity). The cells were stratified based on expression of granzyme-A/granzyme-B/perforin and CD103 (E-cadherin receptor)/CD69 (S1P1 signaling inhibitor)/CD49a (collagen IV receptor). The antibodies used are listed in Supplementary Table 1. Samples were acquired on a 5-laser BD Fortessa-X20 and the results were analyzed by FlowJo software V10.9 (BD Bioscience). Cell frequencies obtained from gates whose parent population was less than 100 cells were excluded from analyses. Due to the constrained quantity of BAL cells and interindividual discrepancies in their CD8 T-cell proportions, we were unable to evaluate all markers for every participant. The exact sample size for each data set is specified accordingly in the Results section. T-distributed stochastic neighbor embedding plots were generated using BAL CD3 + lymphocytes pooled from four study groups (*n* = 5 participant per group; 3905 CD3 + live BAL lymphocytes per group; 15,620 cells in total). Distinct CD8 T-cell populations were identified using FlowSOM, a visualization tool that clusters cells based on chosen flow cytometry markers. FlowSOM arranges cell clusters via a Self-Organizing Map in a Minimum Spanning Tree and groups similar clusters into metaclusters – what we refer to as cell populations [58].

### Statistical analyses

Data were analyzed using GraphPad Prism V10 (San Diego, CA). For all categorical variables, Fisher’s exact test was used. For all quantitative variables, the Kolmogorov-Smirnov test was first used to determine the distribution of variables. The Kruskal-Wallis test was then used to determine significant differences between more than two study groups. Wilcoxon matched-pairs signed rank test and Mann-Whitney rank-sum test were used to compare paired and unpaired study variables respectively. The figures depict only statistically significant *p*-values (* *p* < 0.05, ** *p* < 0.01, *** *p* < 0.001, **** *p* < 0.0001).

## Results

### Participant characteristics

Most of the study participants were male Caucasians (Table 1). PLWH were slightly older compared to uninfected control donors (median age 56 vs. 40 years old). All participating PLWH were under ART with suppressed viral loads and stable CD4 T-cell counts (median: 531 cells/mm3). The final sample size per experiment varied based on the BAL cell viability and cell count after thawing. Details for each experiment performed per sample can be found in Supplementary Table 2.

**Table 1:** Participant Characteristics

Characteristics	*N* = 66				*p* value
	HIV-NS: *N* = 20	HIV-SM: *N* = 11	HIV + NS: *N* = 21	HIV + SM: *N* = 14	
**Demographic factors**					
Age (yrs; median [IQR])	40 [28, 54]	42 [37, 57]	53 [52, 59]	56 [47, 59]	***0.03*** ^1^
Male sex (*n* [%])	20 [100%]	9 [81.8%]	19 [90.5%]	13 [92.9%]	HIV + vs. HIV-: ns^2^ SM vs. NS: ns^2^
***Ethnicity (n [%])***					
White	16 [80%]	11 [100%]	17 [81%]	14 [100%]	ns^2^
Asian	3 [15%]	0 [0%]	0 [0%]	0 [0%]	
Black/Caribbean	0 [0%]	0 [0%]	1 [4.8%]	0 [0%]	
Black/African	0 [0%]	0 [0%]	3 [14.3%]	0 [0%]	
Latino	1 [5%]	0 [0%]	0 [0%]	0 [0%]	
**HIV and immune-related factors**					
Duration of HIV infection (yrs; median [IQR])	N/A	N/A	15 [10, 20]	17 [7, 25]	ns^3^
Duration of time since viral load suppressed (yrs; median [IQR])	N/A	N/A	9 [4, 12]	10 [3, 13]	ns^3^
***Antiretroviral regimen components (n [%])***					
NRTI	5 [25%]	5 [45.5%]	19 [90.5%]	13 [92.9%]	HIV-NS vs. HIV-SM: ns^2^ HIV + NS vs. HIV + SM: ns^2^
N-NRTI	0 [0%]	0 [0%]	5 [23.8%]	6 [42.9%]	
Integrase inhibitor	0 [0%]	0 [0%]	13 [61.9%]	8 [57.1%]	
PI	0 [0%]	0 [0%]	4 [19%]	2 [14.3%]	
INSTI	0 [0%]	0 [0%]	0 [0%]	1 [7.1%]	
Fusion Inhibitor	0 [0%]	0 [0%]	1 [4.8%]	0 [0%]	
CD4 count (cells/mm^3^; median [IQR])	674.5 [518.5, 895]	1069 [920.5, 1210.5]	501 [429, 580]	650.5 [406.75, 811.25]	HIV + vs. HIV-: ***0.003***^3^ SM vs. NS: ***0.02***^3^
CD4/CD8 ratio (median [IQR])	1.8 [1.5, 2.5]	1.8 [1.6, 2.6]	0.7 [0.5, 0.9]	0.9 [0.5, 1.6]	HIV + vs. HIV-: ***<0.0001***^3^ SM vs. NS: ns^3^
**Lifestyle factors**					
Cannabis user (*n* [%])	1 [5%]	5 [45.5%]	1 [4.8%]	9 [64.3%]	HIV + vs. HIV-: ns^2^ SM vs. NS: ***<0.0001***^2^

Results are shown as median and interquartile range (IQR). ^1^Kruskal-Wallis test, ^2^Fisher’s exact test, ^3^Mann–Whitney rank-sum test

### HIV and smoking are independently associated with increased BAL CD8 T-cell frequencies

**Fig. 1:**
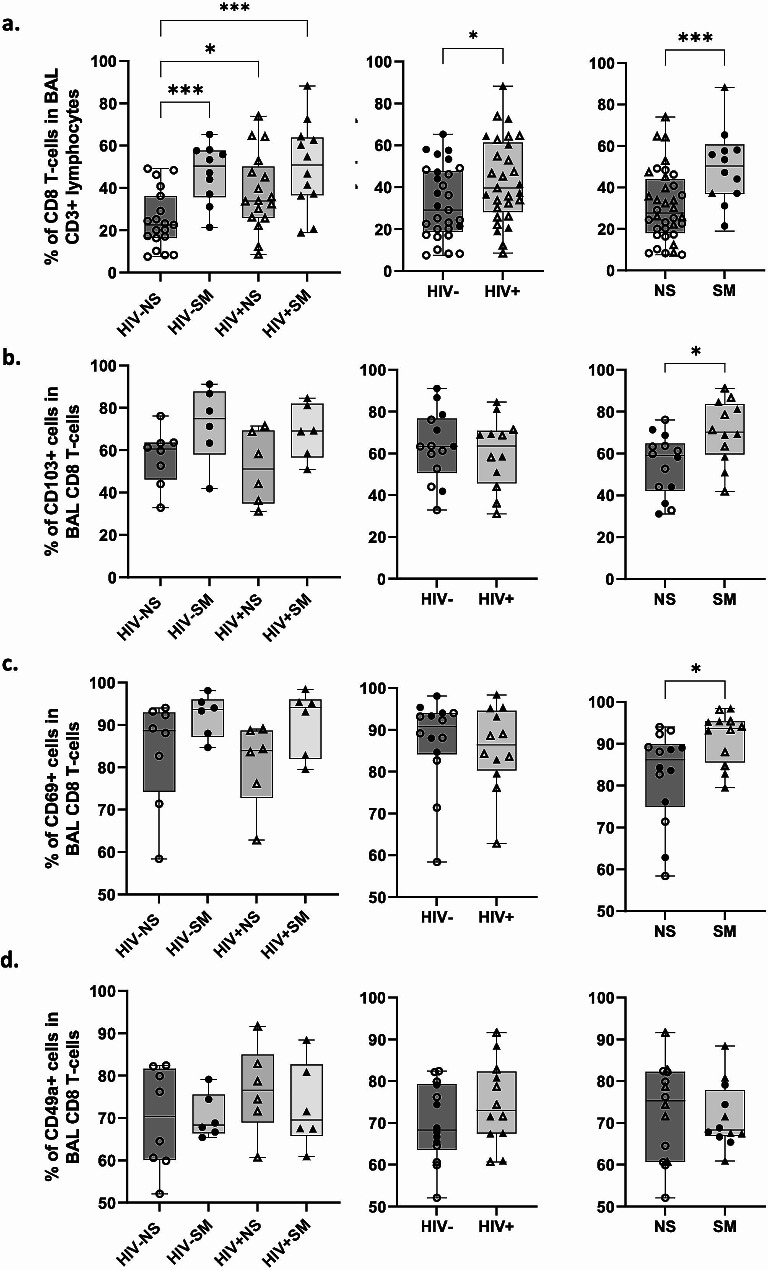
Impact of chronic HIV infection and smoking on BAL CD8 T-cell frequencies and the expression of Trm markers. **(a)** Frequencies of CD8 + CD4- cells in total BAL CD3 + live lymphocytes are shown. **(b-d)** Expression levels of CD103, CD69, and CD49a were assessed within the total CD8 T-cells pool in BAL (HIV-NS: *n* = 8; HIV-SM: *n* = 6; HIV + NS: *n* = 6; HIV + SM: *n* = 6). Data points were stratified by smoking status (right), HIV status (middle), or both (left). Comparisons were made using Mann–Whitney rank-sum test (* *p* < 0.05, ** *p* < 0.01, *** *p* < 0.001, **** *p* < 0.0001)

To evaluate the differences in CD8 T-cell proportions between study groups, BAL CD3 + live singlets expressing the CD8 co-receptor, but not CD4, were gated and their frequencies evaluated within the total live CD3 + lymphocyte pool (Supplementary Fig. 1a). Both smoking and positive HIV status were independently associated with significantly higher BAL CD8 T-cell frequencies (Fig. 1a).

### The majority of BAL CD8 T-cells are tissue-resident

**Fig. 2:**
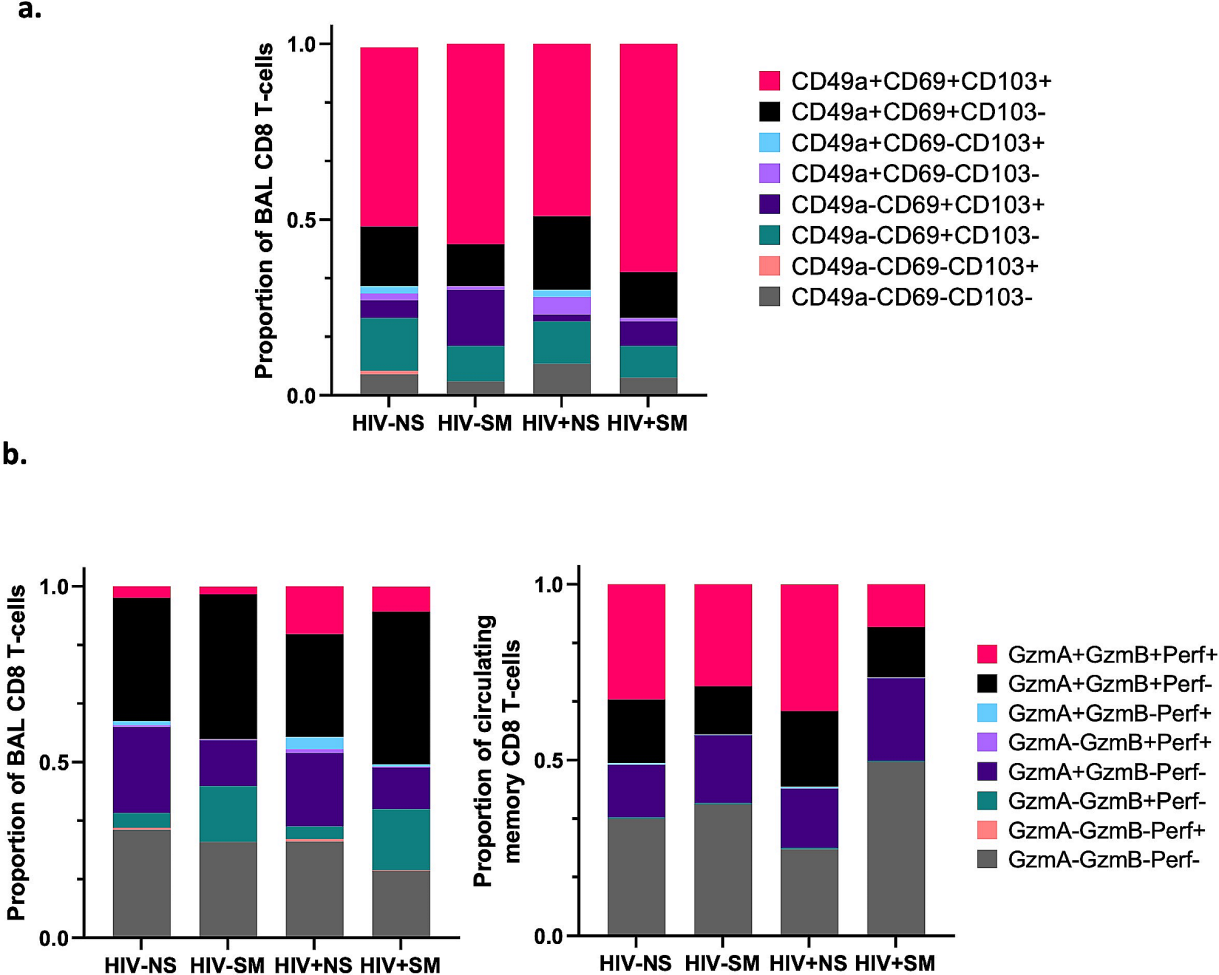
Distribution of Trm subsets and cytotoxic effector subsets among total BAL CD8 T-cells. **(a)** Trm subsets stratified by CD103/CD69/CD49a expression in BAL based on median cell population frequencies in each study group. **(b)** Cytotoxic effector CD8 T-cell subsets stratified by GzmA/GzmB/Perforin expression in BAL and blood based on median cell population frequencies in each study group. (HIV-NS: *n* = 8; HIV-SM: *n* = 6; HIV + NS: *n* = 6; HIV + SM: *n* = 6)

To have a broader view of BAL CD8 T-cell dynamics and effector function, we quantified expression levels of tissue-residency markers (CD103, CD69, CD49a) and effector proteins (GzmA, GzmB, Perforin) in this cell population (Supplementary Fig. 1b). Across all study groups, nearly all BAL CD8 T-cells were positive for at least one Trm marker (Supplementary Fig. 1c, Figs. 1b-d and 2a). Among these populations, the most prominent were CD69 + subsets. Median cell frequencies within total CD8 T-cells are as follows: CD103 + CD69 + CD49a+ (median: 54%), CD103-CD69 + CD49a+ (median: 15%), CD103 + CD69 + CD49a- (median: 6%), CD103-CD69 + CD49a- (median: 11%) and the CD8 non-Trm subset CD103-CD69-CD49a- (median: 5.50%). Frequencies of the remaining CD69- CD8 T-cell subsets were very low (median ≤ 1% per subset). The majority of BAL CD8 T-cells were also positive for GzmA and/or GzmB, with low frequencies of Perforin + cells (Fig. 2b).

It’s worth mentioning that many of the experiments were conducted during the COVID-19 pandemic, a time when bronchoscopies were not allowed, which restricted us to only using frozen samples. During that time, we examined the effect of cryopreservation using matched fresh and frozen peripheral blood mononuclear cells (HIV-NS: *n* = 2) on CD8 T-cell subset markers measured in our flow cytometric panels (Trm: CD103/CD69/CD49a; cytotoxicity: GzmA/GzmB/Perforin, memory: CD45RA/CCR7). We observed no considerable differences between fresh and frozen samples for the exception of one marker – CD69 – that showed a mean increase of 18.83% on CD8 T-cells after cryopreservation (Supplementary Table 3). Although we observed a decrease in live CD3 + BAL lymphocytes, no substantial change in BAL CD8 T-cell viability was observed in frozen *versus* fresh samples (Supplementary Table 4). To verify whether T-cell viability could confound our results, we performed Spearman’s rank correlation analysis and observed no significant relationship between levels of CD69 and T-cell viability (Supplementary Fig. 3).

### Smokers display higher frequencies of CD103+, CD69 + and GzmB + CD8 T-cells in BAL along with increased CD8 T-cell recruitment within pulmonary mucosa

**Fig. 3:**
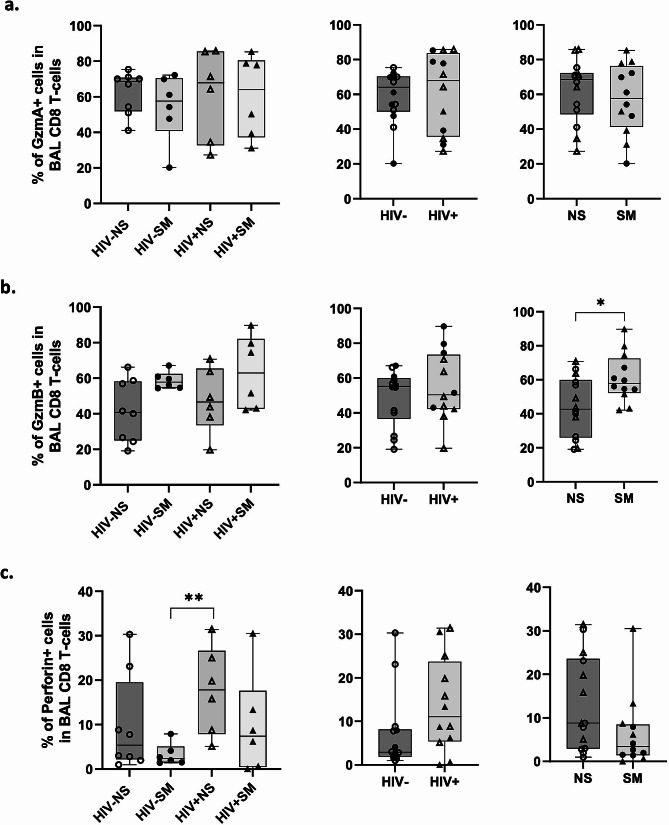
Expression of CD8 T-cell cytotoxic effector molecules GzmA/B and perforin among study groups. Expression levels of GzmA **(a)**, GzmB **(b)**, and Perforin **(c)** were assessed within the total CD8 T-cell pool in BAL (HIV-NS: *n* = 8; HIV-SM: *n* = 6; HIV + NS: *n* = 6; HIV + SM: *n* = 6). Data points were stratified by smoking status (right), HIV status (middle), or both (left). Comparisons were made using Mann–Whitney rank-sum test (* *p* < 0.05, ** *p* < 0.01, *** *p* < 0.001, **** *p* < 0.0001)

**Fig. 4:**
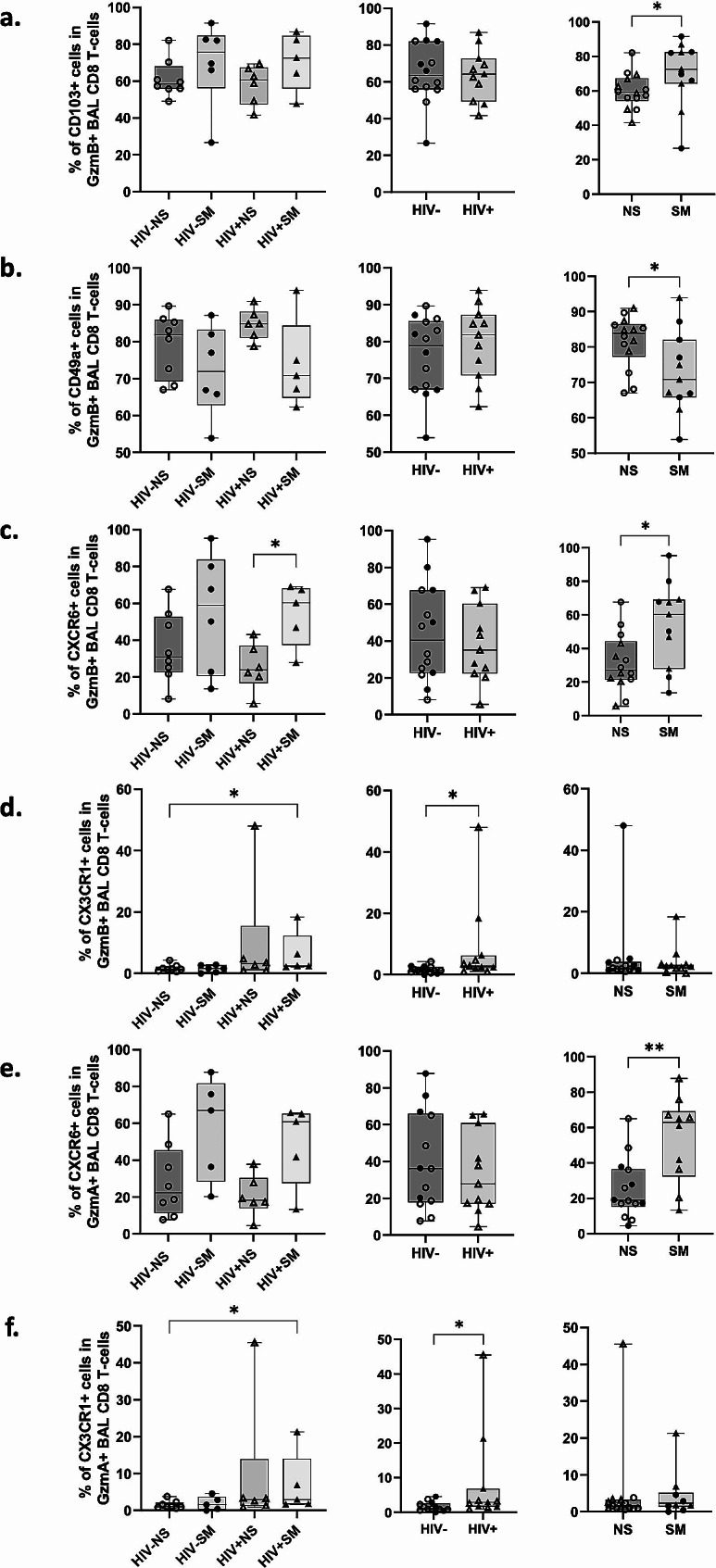
Increased migration and retention of GzmB + CD8 Trm from the interstitium to alveolar space in smokers. **(a-d)** Expression levels of CD103, CD49a, CXCR6, and CX3CR1 in GzmB + BAL CD8 T-cells (HIV-NS: *n* = 8; HIV-SM: *n* = 6; HIV + NS: *n* = 6; HIV + SM: *n* = 5), **(e-f)** as well as CXCR6 and CX3CR1 in GzmA + BAL CD8 T-cells (HIV-NS: *n* = 8; HIV-SM: *n* = 5; HIV + NS: *n* = 6; HIV + SM: *n* = 5) are shown. Data points were stratified by smoking status (right), HIV status (middle), or both (left). Comparisons were made using Mann–Whitney rank-sum test (* *p* < 0.05, ** *p* < 0.01, *** *p* < 0.001, **** *p* < 0.0001)

Only smoking, but not HIV status, was associated with higher frequencies of CD103+, CD69+ (Fig. 1b, c) and GzmB + CD8 T-cells in BAL, while GzmA and perforin expression remained unchanged based on smoking and HIV status (Fig. 3a-c). Since only GzmB + CD8 T-cells are increased in smokers and because GzmB + CD8 T-cells have been associated with COPD severity [59], we further gated on GzmB + cells in our analyses to measure their expression of markers of tissue-residency (CD103, CD69, CD49a), transepithelial migration (CXCR6, CXCR3) [55, 56], proliferative capacity (Ki67), and peripheral infiltration (CX3CR1, KLRG1) [57]. Among smoking participants, GzmB + BAL CD8 T-cells displayed significantly higher levels of CD103 and CXCR6 along with significantly reduced frequencies of CD49a + cells compared to non-smokers (Fig. 4a-c), suggesting increased transepithelial migration and retention of interstitial CD8Trm in the airway lumen. Similarly, significantly higher levels of CXCR6 were observed in GzmA + cells in smokers (Fig. 4e). Notably, in PLWH, we observed significantly higher levels of CX3CR1 on both GzmB + and GzmA + BAL CD8 T-cells (Fig. 4d, f) with a few participants exhibiting particularly elevated levels. Because CX3CR1 is exclusively expressed by non-tissue resident cells coming from the periphery [60], these data suggest increased recruitment of circulating effector CD8 T-cells into the airways in some PLWH. Lastly, we found no significant differences in levels of CXCR3, CX3CR1, KLRG1, and Ki-67 expression among study groups (Supplementary Table 5).

### FlowSOM analysis reveals five distinct BAL CD8 T-cell populations that vary by smoking status

**Fig. 5:**
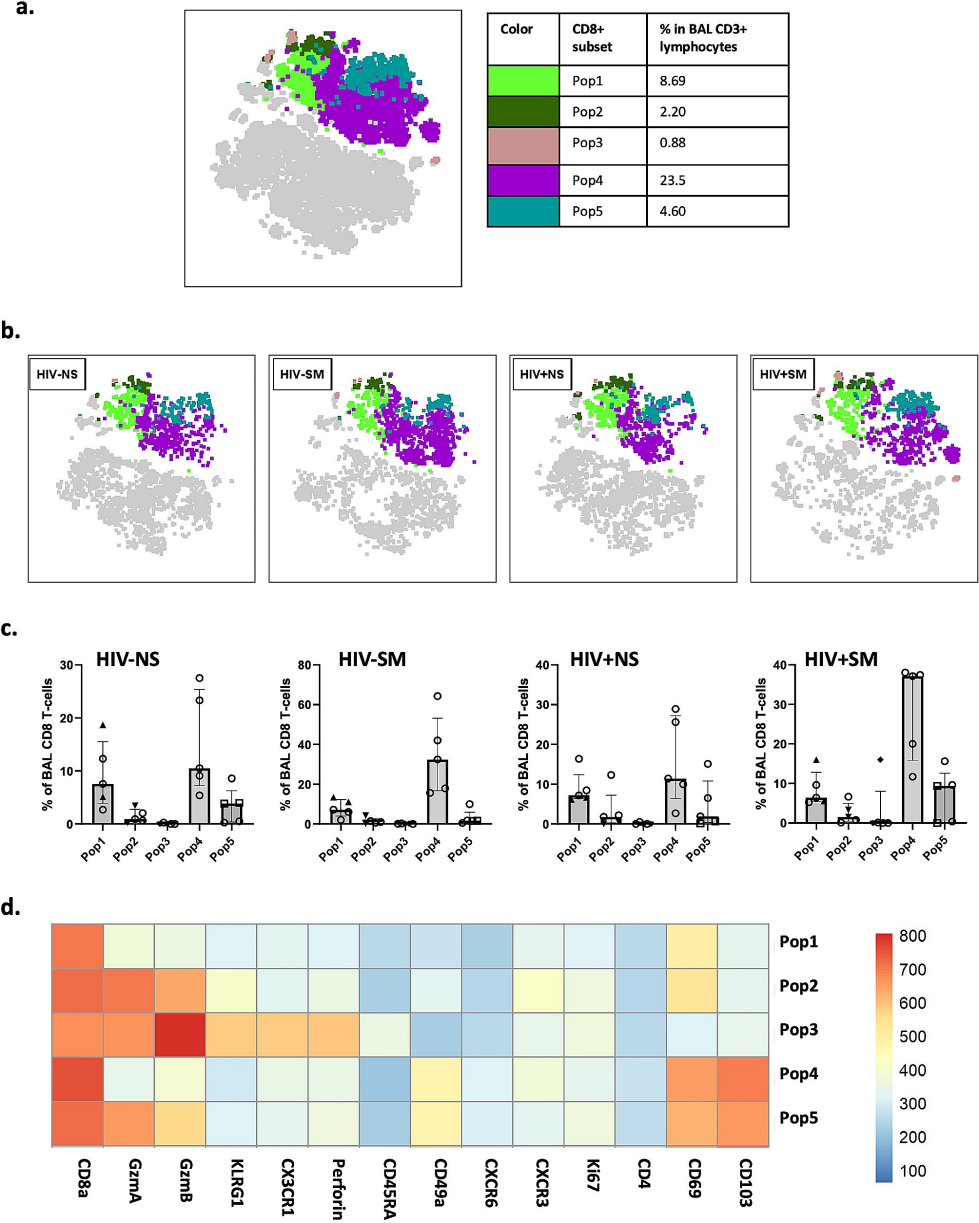
FlowSOM analysis reveals five distinct BAL CD8 T-cell populations that vary by smoking status. **(a)** t-distributed stochastic neighbor embedding (tSNE) plot of sub-population in BAL CD3 + lymphocytes pooled from four study groups (*n* = 5 per group; 3905 CD3 + live BAL lymphocytes per group; 15,620 cells in total). Five different CD8 T-cell populations identified using FlowSOM clustering and visualization technique are shown. **(b)** tSNE plots of BAL CD8 T-cell populations stratified by either HIV, smoking status, or both with **(c)** their corresponding cell frequencies shown as bar plots. **(d)** The heat map displays relative expression of markers used for the FlowSOM analysis in each population identified

To better identify and describe BAL CD8 T-cell subsets distributions, we generated a t-distributed stochastic neighbor embedding (tSNE) plot on BAL CD3 + lymphocytes pooled from four study groups and identified 5 distinct populations using FlowSOM clustering and visualization technique (Fig. 5). In line with our afore mentioned observations, an apparent divide in expression of markers associated with tissue-resident (CD103, CD69, CD49a in Populations 1, 2, 4 and 5) and non-tissue resident (CX3CR1, KLRG1 in Population 3) cells was observed. Among the populations expressing tissue-residency markers, distinct differences in levels of CD103 and GzmA/B are also present. Lastly, we observed significantly higher frequencies of cells in Population 4 in smokers *versus* non-smokers (NS median = 10.95; SM median = 34.75; *p-value* = 0.009), which expressed intermediate levels of GzmB and high levels of CD103, both of which were found to be significantly increased in smoking participants in our previous analyses. Interestingly, this population displayed low levels of GzmA compared to other populations. To confirm this, we performed additional analyses of CD8 T-cell effector subsets based on their combined expression of GzmA/GzmB/Perforin and compared their frequencies between different study groups (Supplementary Fig. 2). Among the GzmB + subsets, only GzmA-GzmB + Perforin- cell frequencies were significantly higher in smokers *versus* non-smokers – a trend that was consistent regardless of HIV status.

### Reduction in naïve and terminally differentiated BAL CD8 T-cells subsets in smokers

**Fig. 6:**
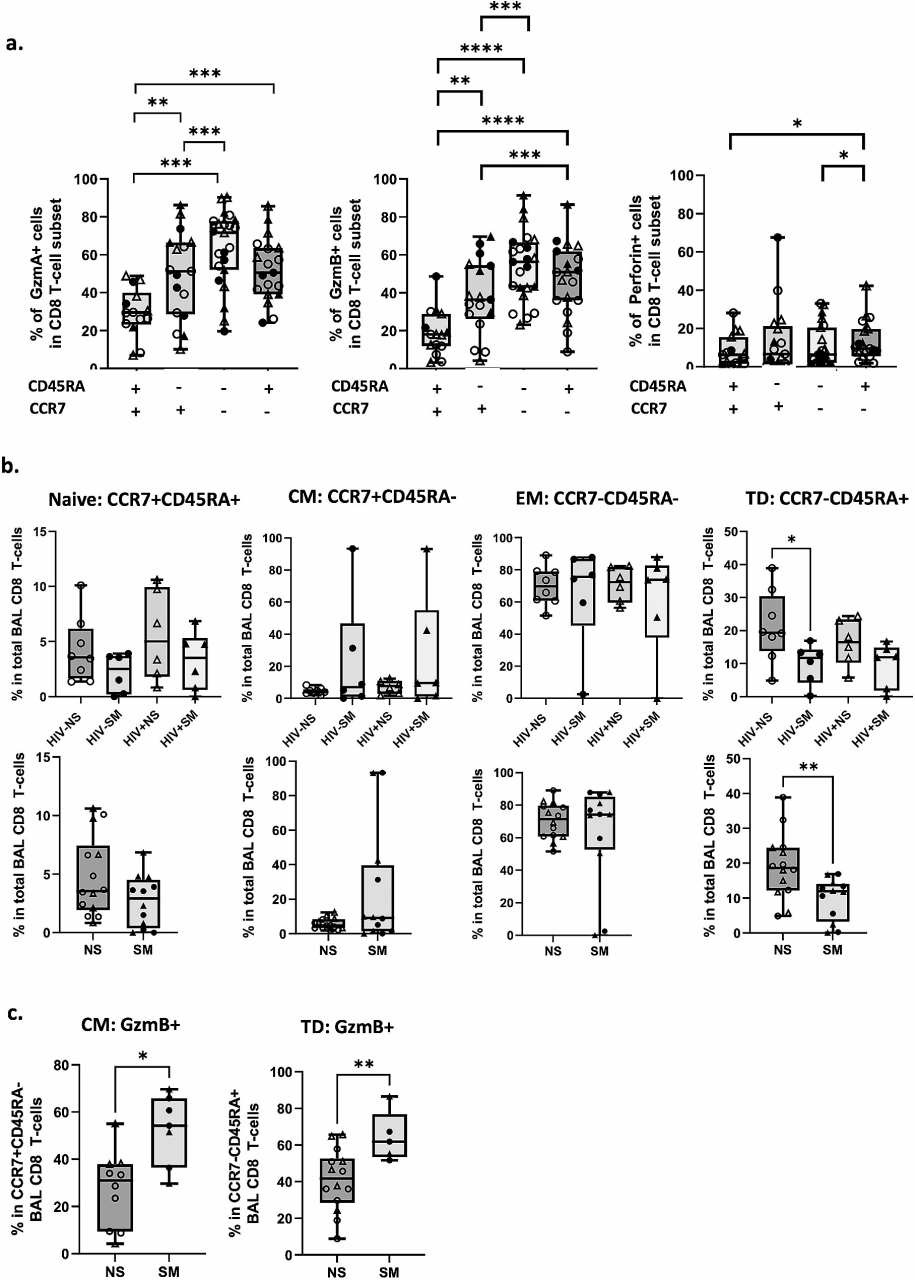
Significantly higher levels of GzmB in effector memory (EM) and terminally differentiated (TD) BAL CD8 T-cells cells in smokers. **(a)** Expression levels of GzmA, GzmB, and Peforin in BAL CD8 T-cell memory subsets (Naïve/ CCR7 + CD45RA+: HIV-NS *n* = 6, HIV-SM *n* = 2, HIV + NS *n* = 5, HIV + SM *n* = 1; central memory (CM) / CCR7 + CD45RA-: HIV-NS *n* = 6, HIV-SM *n* = 4, HIV + NS *n* = 4, HIV + SM *n* = 3; effector memory (EM) CCR7-CD45RA-: HIV-NS *n* = 8, HIV-SM *n* = 5, HIV + NS *n* = 6, HIV + SM *n* = 4), terminally differentiated (TD) CCR7-CD45RA+: HIV-NS *n* = 8, HIV-SM *n* = 3, HIV + NS *n* = 6, HIV + SM *n* = 2; **(b)** Distributions of BAL CD8 T-cell memory subsets between different study groups (HIV-NS: *n* = 8; HIV-SM: *n* = 6; HIV + NS: *n* = 6; HIV + SM: *n* = 6); **(c)** Frequencies of GzmB + cells in CM (left) and TD (right) BAL CD8 T-cell subsets stratified by participant smoking status. Wilcoxon matched-pairs signed rank test and Mann-Whitney rank-sum test were used to compare paired and unpaired study variables respectively (* *p* < 0.05, ** *p* < 0.01, *** *p* < 0.001, **** *p* < 0.0001)

We have previously reported significantly higher levels of CD45RA-CD28 + BAL CD8 T-cells in smokers *versus* non-smokers [30]. Given that we observed a significant increase in GzmB + BAL CD8 T-cells in smokers, we aimed to elucidate whether this increase is linked to cell differentiation stage, since more differentiated cells are known to have more pronounced effector functions [61]. We found that effector memory (EM; CCR7-CD45RA-) and terminally differentiated (TD; CCR7-CD45RA+) cells harbored the highest frequencies of GzmA and GzmB positive cells, with highest levels of perforin found in the CCR7-CD45RA + cell subset (Fig. 6a). Among cross-group comparisons, we have observed a reduction in naïve (N; CCR7 + CD45RA+) and TD BAL CD8 T-cell subsets in smoking *versus* non-smoking participants (Fig. 6b) with a similar skew in proportions toward central memory (CM; CCR7 + CD45RA-). Furthermore, we found that both CM and TD cells have significantly higher levels of GzmB in smokers *versus* non-smokers (Fig. 6c).

## Discussion

Herein, we provide a comprehensive in-depth flow cytometric description of airway CD8 T-cells encompassing their tissue-residency characteristics, effector proteins, markers of migration, and differentiation. We report that HIV and smoking status have distinct and independent effects on lung mucosal CD8 T-cell dynamics. We have found that, while both HIV and smoking are independent factors that can promote higher CD8 T-cell frequencies in BAL, they do so through different mechanisms. Our results suggest that smoking promotes increased migration of GzmA + and GzmB + CD8 T-cells via CXCR6 and augmented retention in the pulmonary mucosa through CD103. Conversely, HIV promotes their infiltration from the periphery via the fractalkine receptor CX3CR1. A significant increase in GzmB + cells in smokers *versus* non-smokers was evident in CM (CCR7 + CD45RA-) and TD (CCR7-CD45RA+) cell subsets. Importantly, among all GzmB + CD8 T-cells, smoking was associated with enrichment of the GzmA-GzmB + Perforin- CD8 T-cell population only.

While CXCR6 is expressed under normal homeostatic conditions in lung CD8 T-cells, it is upregulated substantially during inflammatory responses in context of acute lung infections, chronic lung disease, and aging [62–65]. In context of smoking, increased migration and retention of CD8 T-cells armed with effector proteins like GzmB could be one of the contributors towards increased risk of COPD development in smokers and its subsequent tissue-destructive clinical presentation. Previously, we have reported that, unlike in the blood, BAL CD8 T-cells display high levels of degranulation marker CD107a [30]. Combined with our current observation of enriched GzmB + cells in smokers, collectively this could lead to accumulation GzmB in the extracellular space. Notably, GzmB is a serine protease capable of cleaving extracellular matrix proteins, which could contribute to its degradation and, subsequently, emphysema observed in COPD patients [59]. Our finding that smoking leads to increased levels of GzmB + CD8 T-cells in the lung is in line with previous studies documenting increased CD8 T-cell cytotoxicity in the blood and airways of smokers [66–68]. Furthermore, our observation of increased retention of these GzmB + cells via CD103 is consistent with a recent study by Corleis et al., which reported an increase in CD8 T-cell frequencies in endobronchial brushings of HIV-1 infected smokers [69]. We further show that this is likely caused by increased recruitment of lung interstitial CD8 Trm to the alveolar space via CXCR6, which has also been implicated in COPD and lymphocytic alveolitis [70, 71]. Notably, Freeman et al., have reported that increased CXCR6 expression by CD8 T-cells is positively correlated with COPD severity, meaning that CXCL16/CXCR6 axis blockade might constitute a new therapeutic approach for mitigating some of the damage seen in smoking COPD patients [64].

Significantly higher GzmB levels in CM CD8 T-cells in smokers along with a skewed trend in CD8 memory phenotype from TD toward CM, and the lack of a significant increase in Ki-67 could be indicative of increased cellular trafficking (rather than homeostatic proliferation) of younger memory cells into the alveolar space, and simultaneous clearing of older TD cells. While, by definition, CD8 Trm cells do not express CCR7 and we did not observe increased levels of CX3CR1 in smokers, it is does not rule out a potential increase in CD8 T-cell infiltration not only from the interstitial CD8 Trm cell pool but also the CCR7 + CM cell pool from the peripheral circulation. While we used CX3CR1 as a marker for circulating CD8 T-cells, since it is not expressed by tissue-resident cells, some studies have pointed out a distinction between CX3CR1 + and CX3CR1- circulating CD8 T-cell memory subsets [60], leaving open the possibility that lack of CX3CR1 on BAL CD8 T-cells in smokers might indicate that the infiltrating CD8 T-cells are at an earlier differentiation stage.

Considerably elevated levels of CX3CR1 observed in some of our study participants who are PLWH might be linked to the residual lung HIV reservoir that persists despite long-term suppressive ART, which our group and others have reported on previously [13, 72, 73]. Importantly, residual HIV proteins found in BAL fluid of PLWH on ART are capable of mediating T-cell chemotaxis [74, 75]. Existing literature also postulates that CX3CR1 + CD8 T-cells can act as a double-edged sword, contributing to viral control [76] but also increasing the risk of inflammatory diseases in PLWH [77]. Enrichment in CX3CR1 + circulating memory CD8 T-cells in PLWH on ART has been previously reported by Mudd et al. [77]. In their work, the authors argued that the expanded CD8 T-cells in PLWH on ART could preferentially localize to vascular endothelium and contribute to cardiovascular disease onset. Importantly, the CX3CL1-CX3CR1 axis has been implicated in various chronic inflammatory lung diseases, such as COPD and pulmonary fibrosis [78], whose rates are also increased in PLWH on ART [1, 78]. One of the pathogenic mechanisms observed in these chronic lung conditions is CD8 T-cell accumulation within the lung vessel wall and parenchyma [17, 59, 78]. In summary, increased frequencies of BAL CD8 T-cells, mediated through chemokine receptors like CX3CR1, could be one of the culprits behind increased lung disease rates in PLWH on ART.

One of this study’s challenges and subsequent limitations was gathering of sufficient biological material. Because a large portion of the experiments was performed during the COVID-19 pandemic, all bronchoscopies were prohibited, limiting us to frozen samples only. This has significantly impacted cell viability and subsequent cell count for ongoing experiments, which is reflected in the varying sample sizes between data sets. To verify whether the drop in cell viability and recovery had an effect on other markers being measured, we have performed additional tests and analyses comparing fresh and frozen BAL and PBMC samples using our flow cytometric panels after the ban on bronchoscopies was lifted. We observed no prominent changes except for one marker – CD69 – whose frequencies were higher in frozen *versus* fresh CD8 T-cells coming from the same donors. CD69 is known to be increased upon T-cell activation and cell stress, both of which are likely during the heat shock the cells are put under upon thawing [79, 80]. A similar increase in CD69 after cryopreservation has also been previously reported in circulating human NK cells [81]. Collectively, because all BAL samples in this study were cryopreserved and then processed in the same manner, we do not anticipate that this would affect our cross-group comparisons.

## Conclusions

Overall, results of this study provide an additional puzzle piece to the bigger picture of the pulmonary immune system in PLWH on ART and in smokers. We have provided an extensive description of GzmB + CD8 T-cell populations that are enriched in context of smoking and how they compare between PLWH and seronegative controls. We report that the profound effect on CD8 T-cell frequencies in BAL observed in context of smoking and HIV is likely mediated through different migration/homing mechanisms, and significant differences in CD8 T-cell phenotypes between the two groups. Collectively, these findings can help inform further studies exploring new targets to design therapeutics that could help alleviate chronic emphysema and bronchiolitis in PLWH on ART. Importantly, such therapeutic approaches might need to be tailored to smoking status.

### Electronic supplementary material

Below is the link to the electronic supplementary material.

Supplementary Material 1

Supplementary Material 2

## Data Availability

No datasets were generated or analysed during the current study.

## Declarations

### Additional information

Abstracts of data included in the paper have previously been presented at the AIDS 2022 Conference, Montreal, QC, Canada; and, at the IAS 2023 conference, Brisbane, Australia.
