# Supplementary material for: Dynamics of pulmonary mucosal cytotoxic CD8 T-cells in people living with HIV under suppressive antiretroviral therapy
Source: Respir Res. 2024 Jun 12;25:240. doi: 10.1186/s12931-024-02859-2 (PMC11170847; doi:10.1186/s12931-024-02859-2)
Supplement: Supplementary file 1 — Supplementary Material 1 [file 12931_2024_2859_MOESM1_ESM.pdf]

SUPPLEMENTARY MATERIAL

Supplementary Table 1: Flow cytometry reagents used in immunophenotyping experiments.

Marker	Fluorochrome	Clone	Company
**CCR7 (CD197)**	BV510	3D12	BD Horizon, BD Biosciences, San Jose, CA
**CD103**	PerCP-eFluor710	B-Ly7	Invitrogen, Life Technologies Corporation, Eugene, OR
**CD3**	BV 786	SP34-2	BD Horizon, BD Biosciences, San Jose, CA
**CD4**	BV 650	L200	BD Horizon, BD Biosciences, San Jose, CA
**CD45**	PE/Cy7	HI30(RUO)	BD Pharmigen, BD Biosciences, San Jose, CA
**CD45RA**	BUV 737	HI100	BD Horizon, BD Biosciences, San Jose, CA
**CD49a**	APC	TS2/7	BioLegend, San Diego, CA
**CD69**	BV605	Fn50	BD Horizon, BD Biosciences, San Jose, CA
**CD8**	PE-CF594	RPA-T8	BD Horizon, BD Biosciences, San Jose, CA
**CX3CR1**	BV421	2A9-1	BD Horizon, BD Biosciences, San Jose, CA
**CXCR3**	R718	1C6/CXCR3	BD Horizon, BD Biosciences, San Jose, CA
**CXCR6 (CD186)**	BV711	13B 1E5	BD OptiBuild, BD Biosciences, San Jose, CA
**Granzyme A**	PE	CB9	Invitrogen, Life Technologies Corporation, Eugene, OR
**Granzyme B**	PE/Cy7	QA18A28	BioLegend, San Diego, CA
**Ki67**	APC eFluor780	SolA15	Invitrogen, Life Technologies Corporation, Eugene, OR
**KLRG1**	PE-CF594	14C2807	BioLegend, San Diego, CA
**LiveDead**	350nm excitation	N/A	Invitrogen, Life Technologies Corporation, Eugene, OR
**Perforin**	FITC	Pf-344	Mabtech, Stockholm, Sweden

Supplementary Table 2: Experiments and analyses performed for each participant BAL sample.

Participant ID	Study group	Flow cytometry 1: % of CD8 T-cells in total live BAL CD3+ lymphocytes	Flow cytometry 2: In-depth characterization of cell origin, migration, cytotoxicity, and memory (CCR7/CD45RA) subsets
AMC10	HIV-NS	Yes	No
AMC11	HIV-NS	Yes	Yes
AMC12	HIV-NS	No	No
AMC18	HIV-NS	Yes	Yes
AMC19	HIV-NS	Yes	Yes
AMC20	HIV-NS	Yes	Yes
AMC21	HIV-NS	Yes	Yes
AMC25	HIV-NS	Yes	Yes
AMC27	HIV-NS	Yes	Yes
AMC33	HIV-NS	Yes	No
AMC34	HIV-NS	Yes	No
AMC37	HIV-NS	Yes	No
AMC38	HIV-NS	No	No
Patient025	HIV-NS	Yes	No
Patient026	HIV-NS	Yes	No
Patient028	HIV-NS	Yes	No
Patient029	HIV-NS	Yes	No
Patient030	HIV-NS	Yes	No
Patient032	HIV-NS	Yes	No
Patient049	HIV-NS	Yes	No
AMC02	HIV-SM	No	No
AMC09	HIV-SM	Yes	Yes
AMC14	HIV-SM	Yes	No
AMC22	HIV-SM	Yes	Yes
AMC23	HIV-SM	Yes	Yes
AMC28	HIV-SM	Yes	Yes
AMC32	HIV-SM	Yes	Yes
AMC35	HIV-SM	Yes	No

AMC36	HIV-SM	Yes	No
Patient033	HIV-SM	No	No
Patient053	HIV-SM	Yes	Yes
AMV03	HIV+NS	Yes	Yes
AMV15	HIV+NS	Yes	Yes
AMV22	HIV+NS	No	No
AMV25	HIV+NS	Yes	Yes
AMV26	HIV+NS	Yes	Yes
AMV27	HIV+NS	Yes	Yes
Patient010	HIV+NS	Yes	No
Patient014	HIV+NS	Yes	No
Patient016	HIV+NS	No	No
Patient021	HIV+NS	Yes	No
Patient023	HIV+NS	Yes	No
Patient024	HIV+NS	Yes	No
Patient037	HIV+NS	Yes	No
Patient039	HIV+NS	Yes	No
Patient041	HIV+NS	Yes	No
Patient043	HIV+NS	No	No
Patient045	HIV+NS	No	No
Patient046	HIV+NS	No	No
Patient048	HIV+NS	Yes	No
Patient050	HIV+NS	No	No
Patient055	HIV+NS	Yes	Yes
AMV04	HIV+SM	Yes	No
AMV11	HIV+SM	No	No
AMV16	HIV+SM	No	No
AMV17	HIV+SM	Yes	Yes
AMV18	HIV+SM	Yes	No
AMV19	HIV+SM	Yes	No
AMV21	HIV+SM	No	No
AMV23	HIV+SM	Yes	Yes
Patient038	HIV+SM	Yes	No

Patient040	HIV+SM	No	No
Patient047	HIV+SM	Yes	No
Patient051	HIV+SM	Yes	Yes
Patient052	HIV+SM	Yes	Yes
Patient054	HIV+SM	Yes	Yes

Supplementary Table 3: Effect of cryopreservation on CD8 T-cell subset markers in fresh vs frozen peripheral blood mononuclear cells (HIV-NS: *n=2*).

Marker	Mean % in fresh cells	Mean % in frozen cells	Mean % in frozen - Mean % in fresh	Fold change
**% live cells in single CD3+ circulating lymphocytes**	97.65	96.45	-1.20	-0.01
**%CD69 in CD8 T-cells**	4.64	23.47	18.83	4.06
**%CD103 in CD8 T-cells**	4.21	1.88	-2.33	-0.55
**%CD49a in CD8 T-cells**	1.85	1.08	-0.77	-0.42
**%Granzyme A in CD8 T-cells**	25.75	22.04	-3.71	-0.14
**%Granzyme B in CD8 T-cells**	15.10	14.32	-0.78	-0.05
**%Perforin in CD8 T-cells**	12.39	10.39	-2.00	-0.16
**%CD45RA in CD8 T-cells**	68.90	71.45	2.55	0.04
**%CCR7 in CD8 T-cells**	50.40	47.55	-2.85	-0.06

Supplementary Table 4: Effect of cryopreservation on cell viability in fresh vs frozen BAL CD3+ and CD8+ T-cells (HIV-NS: *n=1*; HIV+SM: *n=1*).

Marker	Mean % in fresh circulating cells	Mean % in frozen circulating cells	Mean % in frozen - Mean % in fresh	Fold change
**% live cells in total BAL CD3+ lymphocytes**	92.9	51.7	-41.20	-0.44
**% of CD8 T-cells in total live BAL CD3+lymphocytes**	45.7	46.45	0.75	0.02

Supplementary Table 5: Additional flow cytometry analyses in GzmA+/GzmB+ BAL CD8 T-cells.

**BAL CD8 T-cell Population**	**Study group**				
	**HIV-NS** (n=8)[1]	**HIV-SM** (n=6)[1]	**HIV+NS** (n=6)[1]	**HIV+SM** (n=5)[1]	**p-value**[2]
**In GzmA+ BAL CD8**					
**CXCR3+**	69.15 (64.05, 72.48)	82.9 (59.2, 83.3)	52.7 (15.7, 60.4)	56 (53.1, 76)	0.5
**CX3CR1+**	1.01 (0.91, 1.79)	1.58 (1.43, 2.86)	2.92 (1.68, 3.37)	2.95 (1.77, 6.86)	0.12
**KLRG1+**	20.3 (6.19, 33.65)	7.35 (6.96, 18.4)	14.65 (6.29, 19.25)	27.6 (15.6, 32.7)	0.8
**Ki67+**	2.42 (1.68, 3.15)	4.66 (1.31, 4.79)	4.37 (3.82, 5.95)	3.03 (2.7, 3.77)	0.3
**In GzmB+ BAL CD8**					
**CXCR3+**	73.15 (65.13, 76.58)	83.3 (66.95, 85.93)	51.9 (14, 63.4)	59.1 (53.2, 84.2)	0.5
**KLRG1+**	21.65 (6.08, 36.48)	8.78 (6.25, 13.7)	10.83 (5.54, 20.6)	21.5 (21.1, 28.1)	0.8
**Ki67+**	4.24 (2.38, 5.11)	3.23 (1.77, 5.54)	4.88 (4.65, 5.62)	2.47 (2.05, 2.74)	0.2

[1] Median of % subset frequencies (IQR);
[2] Kruskal-Wallis Rank Sum test
