# Supplementary material for: Dynamics of pulmonary mucosal cytotoxic CD8 T-cells in people living with HIV under suppressive antiretroviral therapy
Source: Respir Res. 2024 Jun 12;25:240. doi: 10.1186/s12931-024-02859-2 (PMC11170847; doi:10.1186/s12931-024-02859-2)
Supplement: Supplementary file 2 — Supplementary Material 2 [file 12931_2024_2859_MOESM2_ESM.pdf]

Supplementary Figure 1

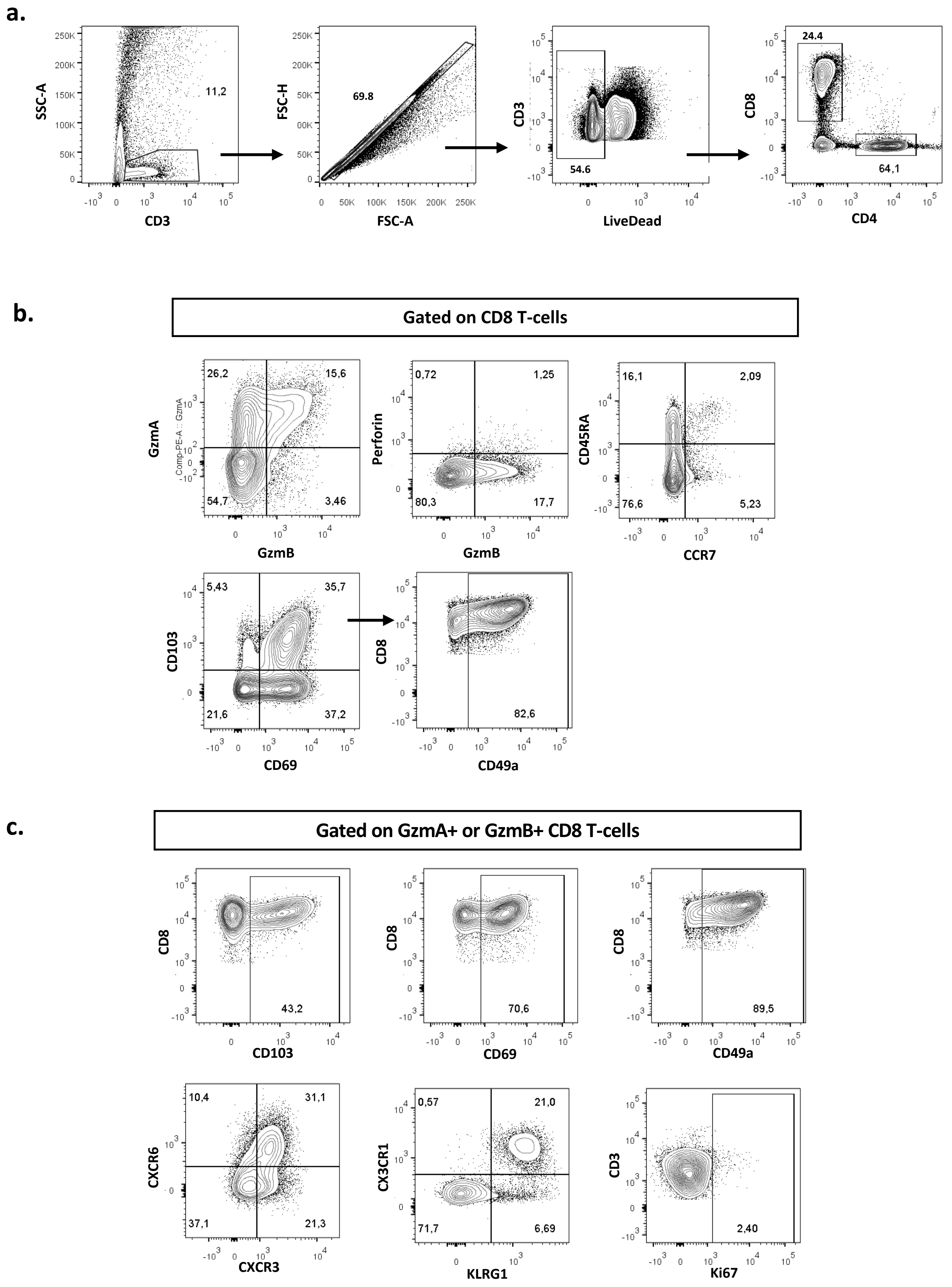

Supplementary Figure 1: Gating strategy used for flow cytometry analysis. (a) CD8 T-cells were defined as CD3+ live singlets expressing the CD8 co-receptor but not CD4. **(b)** Frequencies of cells expressing effector proteins (GzmA/GzmB/Perforin), markers of tissue-retention (CD103/CD69/CD49a), and memory subsets (CCR7/CD45RA/CD28) within the total CD8 T-cell pool were assessed. **(c)** To investigate the phenotype of armed effector CD8 T-cells, GzmA+ or GzmB+ CD8 T-cell populations were gated and analyzed for expression of tissue-residency markers (CXCR3/CXCR6, CD103/CD69/CD49a), blood-origin markers (CX3CR1/KLRG1), and proliferative potential (Ki67).

Supplementary Figure 2

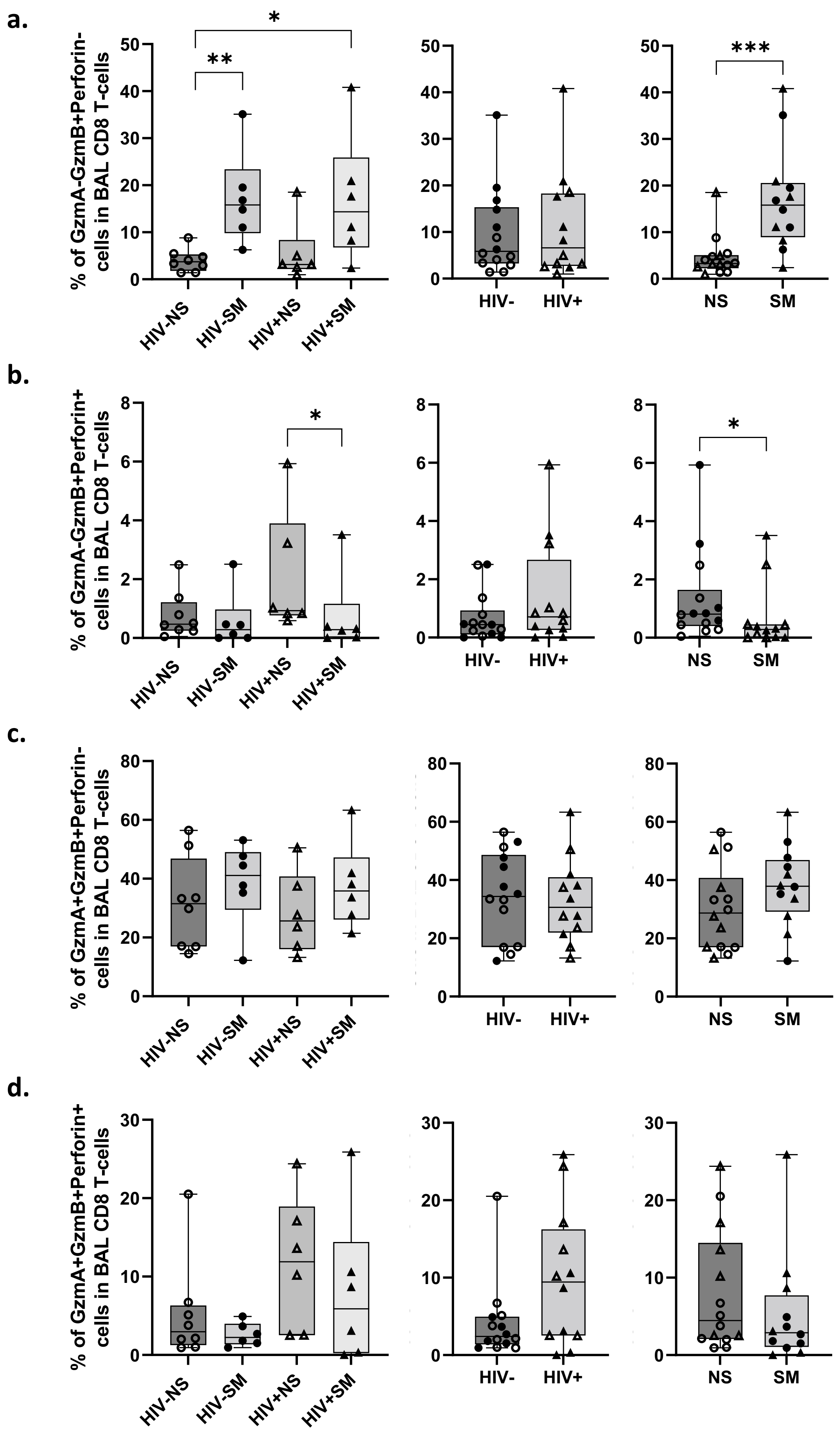

Supplementary Figure 2: GzmB+ CD8 T-cells increased in smokers are negative for GzmA and Perforin. (a-d) Frequencies of BAL CD8 T-cell subsets based on their combined expression of GzmA/GzmB/Perforin were assessed to determine which GzmB+ CD8 T-cell subsets were enriched in smoking participants (HIV-NS: *n=8*; HIV-SM: *n=6*; HIV+NS: *n=6*; HIV+SM: *n=6*). Data points were stratified by HIV status (**middle**), smoking status (**right**), or both (**left**). Comparisons were made using Mann–Whitney rank-sum test (* $p < 0.05$, ** $p < 0.01$, *** $p < 0.001$, **** $p < 0.0001$).

Supplementary Figure 3

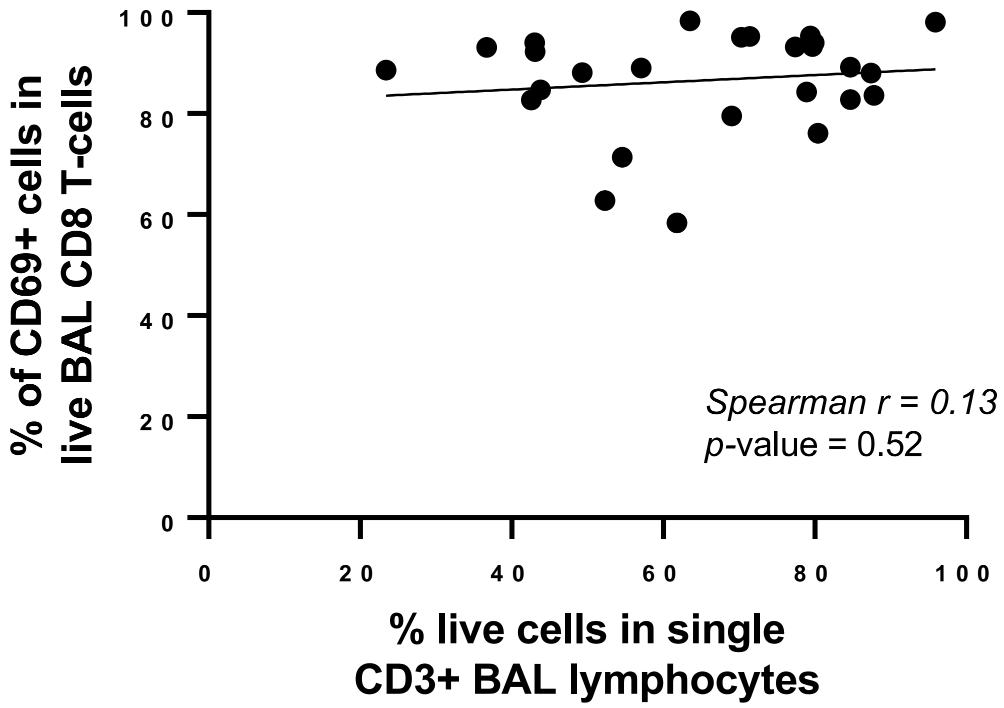

Supplementary Figure 3: Correlation between T-cell viability and CD69 expression in CD8 T-cells in BAL. Results of Spearman's rank correlation between T-cell viability and % of CD69+ CD8 T-cells in BAL are shown (HIV-NS: *n=8*; HIV-SM: *n=6*; HIV+NS: *n=6*; HIV+SM: *n=6*).
